# A longitudinal study on the relationships between impulsivity and excessive smartphone use among patients with acquired brain injury and control participants

**DOI:** 10.3389/fpsyt.2025.1691748

**Published:** 2025-11-06

**Authors:** Yehuda Wacks, Meni Koslowsky, Ayala Bloch, Aviv Weinstein

**Affiliations:** Psychology Department, Ariel University, Ariel, Israel

**Keywords:** excessive smartphone use, impulsivity, acquired brain injury (ABI), OFC syndrome, treatment

## Abstract

**Introduction:**

Previous studies have demonstrated that impulsivity is positively correlated with excessive smartphone use, indicating the involvement of frontal lobe circuits. This study examined excessive smartphone use, impulsivity, and mental wellbeing in patients with acquired brain injury (ABI) before and after occupational rehabilitation treatment, and control participants.

**Procedure:**

Participants consisted of 44 patients with ABI [10 patients with orbitofrontal syndrome (OFS) and 34 without OFS] and 69 control participants with no history of brain injury. The procedure included a smartphone application that tracked daily smartphone use and frequency of device unlocks, computerized tasks that evaluated impulsive choice (Delay Discounting Task), impulsive action or response inhibition (the ability to stop an already-initiated action—the Go/No-Go task), and questionnaires measuring excessive smartphone use, obsessive–compulsive symptoms [Yale–Brown Obsessive–Compulsive Scale (YBOCS)], impulsivity [Barratt Impulsiveness Scale (BIS-11), which measures non-planning, motor and attention impulsivity], and mental wellbeing [Depression, Anxiety, and Stress Scale (DASS-21), which measures depression, anxiety, and stress]. Data were collected at two time points: baseline (T1) and 5 months later (T2).

**Results:**

At baseline (T1), patients with ABI and OFS exhibited higher impulsive action, indicated by more commission errors on the Go/No-Go task, excessive smartphone use, and higher ratings of depression compared with control participants. Secondly, patients with ABI without OFS showed higher trait attention-impulsivity ratings compared with control participants. After treatment (T2), patients with ABI showed improved impulsive choice, indicated by improved delay discounting, but no improvement in smartphone use.

**Discussion:**

Brain injury, particularly in frontal regions, is associated with impulsiveness and excessive smartphone use. Patients with ABI showed an improvement in delay discounting after treatment, which is likely due to occupational therapy and training in control of impulsivity. It is recommended that specific treatment program for excessive smartphone use will be developed for patients with ABI.

## 1 Introduction

### 1.1 Excessive smartphone use

In recent years, there has been a significant increase in social media use, particularly through smartphones (1, 2). Excessive smartphone use has been associated with numerous negative effects, such as mental disorders (3, 4), cognitive impairments (5, 6), and impaired function (7, 8). The increasing prevalence of smartphone use and its potential consequences have generated a growing body of research in this domain. Many studies aim to identify factors predicting excessive smartphone use (9) and the specific brain regions and circuits associated with these factors (8, 10). Impulsivity has been identified as a significant predictor of excessive smartphone use (11, 12). Impulsive behavior can stem from early-developing personality traits, known as trait impulsivity (13), or deficits in response inhibition (14). Excessive smartphone use has also been associated with symptoms of obsessive–compulsive disorder (15) and with comorbidity with depression, anxiety, low self-esteem, low psychological wellbeing, and low mental wellbeing (6).

### 1.2 Trait impulsivity and excessive smartphone use

Impulsivity is defined as a behavior that is characterized by decreased sensitivity to negative consequences of behavior; rapid, unplanned reactions to stimuli before complete processing of information; and a lack of regard for long-term consequences (16). Impulsivity involves “actions that are poorly conceived, prematurely expressed, unduly risky, or inappropriate to the situation and that often result in undesirable outcomes” (17). The abilities to regulate impulsivity and inhibit responses are related to the frontal lobe, particularly the prefrontal cortex, and its associated networks (18–20). Recent studies revealed an association between reduced frontal lobe activity and excessive smartphone use (21, 22). Recent brain imaging studies have shown structural alterations in the prefrontal cortex that were related to problematic smartphone use (23, 24).

Prior research has demonstrated an association between trait impulsivity and excessive smartphone use. Grant et al. (25) reported an association between impulsivity, measured by the Barrett Impulsivity Questionnaire, and excessive smartphone use in a sample of 10,000 young individuals in the United States. Similar findings were observed among students in England (11), young individuals in Korea (26), and both young individuals and adults in Germany (27). Additionally, Efrati et al. (28) found a positive association between trait impulsivity and problematic social media use among adolescents. Although self-report measures of impulsivity are useful for assessing various cognitive and behavioral styles (29), they have limitations in objectively characterizing impulsive behavior. Therefore, additional behavioral paradigms have been developed to evaluate specific facets of impulsivity, such as deficiencies in delayed gratification and inhibitory response deficits. It should be clarified that attentional impulsivity refers to the ability to ignore distractions and focus attention, whereas response inhibition is the ability to stop an already-initiated action.

### 1.3 Impulsive choice, delay discounting, and response inhibition

Impulsive decision-making is commonly examined using the temporal discounting paradigm, which involves presenting individuals with a choice between smaller, immediate rewards and larger, delayed rewards. Selecting immediate rewards reflects impulsivity, whereas choosing delayed rewards demonstrates self-control (30). Previous research demonstrated an association between delay discounting and smartphone overuse (21, 31, 32). Deficiencies in inhibitory control can manifest as impulsive behavior (14). This inhibitory control mechanism enables the suppression of irrelevant stimulus responses and ineffective action strategies; however, when compromised, individuals struggle to regulate their behavior despite their intentions to respond appropriately (33). The inhibitory control capacity is commonly measured through the Go/No-Go and Stop Signal Task (SST) paradigms. Deficits in motor inhibitory control have been associated with excessive smartphone use. Chen et al. (34) used the Go/No-Go task and electrophysiological measures (ERPs) to assess response inhibition among smartphone users, finding a negative correlation between excessive smartphone use and response inhibition. Similar findings were reported using the SST (21). These studies have established a clear relationship between response inhibition and excessive smartphone use. As previously mentioned, impulsivity and response inhibition are closely associated with the functioning of the frontal lobes, particularly the prefrontal cortex, and its networks.

Delay discounting is associated with the activity of several brain regions, including the precuneus, the prefrontal cortex, the ventromedial prefrontal cortex, the insula, and the anterior cingulate cortex in human participants in functional magnetic resonance imaging (fMRI) (35). Response inhibition was associated with involvement of the superior medial and right inferior prefrontal cortices during performance of response inhibition tasks in human participants in fMRI (36, 37). The majority of research exploring these associations has focused on healthy individuals without any history of acquired brain injury (ABI). Therefore, the objective of this study was to examine these relationships specifically among individuals with ABI.

### 1.4 Acquired brain injury (ABI)

ABI is defined as brain damage occurring after birth, excluding congenital disorders, developmental defects, or progressive degenerative processes (38). Cognitive impairments in ABI are common, including memory problems and attention difficulties (39). The location of the brain injury often determines the specific deficits (40). The frontal lobe, responsible for diverse behaviors, is particularly susceptible to dysfunction, resulting in difficulties with movement, language, mood, attention, memory, and executive functions (41). The orbitofrontal syndrome (OFS) is a variant of the frontal lobe syndrome, which is associated with behavioral impairments such as hyperactivity and distractibility, and an inability to comply with social rules (42). The OFS is also characterized by a lack of inhibition and impulsivity, often leading to poor social judgment, tactless behavior, and inappropriate actions. Individuals with OFS often show distractibility and difficulties in behavioral control, leading to impulsive behavior (43).

### 1.5 Impulsivity, frontal dysfunction, and smartphone use

Impulsive behavior in individuals with OFS can manifest through difficulty in delaying gratification and impaired inhibitory control. Studies utilizing the temporal discounting paradigm indicate that individuals with frontal brain injuries often prefer immediate, smaller rewards over larger, delayed ones, reflecting impulsive decision-making (31, 44). Another dimension of impulsivity, inhibitory control, is also affected in individuals with ABI, evidenced by difficulties in disinhibition measured by response inhibition tasks such as the Stroop, Go/No-Go, and the SST (45).

### 1.6 Treatment for ABI

Individuals with ABI are treated in a rehabilitation program that is designed to help patients adjust to life after injury, particularly in occupational therapy. The program includes functional rehabilitation, individual and group psychotherapy, cognitive interventions in order to treat the injury and its consequences, vocational preparation, and family guidance to improve mental wellbeing (46, 47). Although there is evidence for the effectiveness of cognitive training after traumatic brain injury (48), it is unknown whether the rehabilitation program is also useful in improving impulsivity, impaired behavioral inhibition, and excessive smartphone use among patients with ABI.

### 1.7 Rationale of the study

Previous studies have established a clear relationship between impulsivity and inhibition, which are associated with frontal lobe function and excessive smartphone use. The majority of research exploring these associations has focused on healthy individuals without any history of ABI. The purpose of this study is to compare measures of impulsivity, mental wellbeing, and excessive smartphone use between individuals with ABI. Most brain injuries are widespread, and they involve many brain regions and networks, and it is very difficult to isolate the injury by region. We included patients with non-specific ABI and those with specific OFS since the OFS group is expected particularly to show impulsivity and response inhibition in comparison with control individuals. Secondly, it will assess whether individuals with ABI and OFS, who are treated in a rehabilitation program, would show reduced impulsivity, response inhibition and excessive smartphone use, and improved mental health after treatment.

### 1.8 Hypotheses

1. Participants with ABI will show higher rates of anxiety, depression, stress, and obsessive–compulsive symptoms compared with healthy participants.

2. Participants with ABI with OFS will exhibit greater impulsivity, as measured by cognitive tasks that test response inhibition and delay discounting and self-report questionnaires, compared with participants with ABI without OFS and healthy participants.

3. Participants with ABI with OFS will exhibit excessive smartphone use, indicated by average daily hours of device use and average daily number of unlocks as measured by a smartphone usage app that measures and ratings on a self-report questionnaire, compared with participants with ABI without OFS and healthy participants.

4. Participants with ABI with OFS who participate in a neuropsychological rehabilitation program will experience a decrease in impulsivity and a decrease in average excessive smartphone use compared to the beginning of treatment.

5. Participants with ABI who participate in a rehabilitation program will experience a decrease in excessive smartphone use over time and an improvement in the level of mental wellbeing compared to the beginning of treatment.

## 2 Method

### 2.1 Participants

Data collection was conducted for 18 months from January 2022 to August 2023. A total of 113 participants participated in the study. The research sample consisted of two groups: a group of 44 individuals who had experienced a brain injury (including 10 participants with evidence of OFS and 34 participants without evidence of OFS) and a control group of 69 individuals with no prior history of brain injury. Recruitment for the control group was carried out through popular social networks such as “Facebook” and “WhatsApp”. The participants with ABI were recruited from the National Institute for Neuropsychological Rehabilitation. All participants in the study participated voluntarily.

Participants’ demographic data and drug use history are described in **Table 1**. The mean age of control participants was 27 years and 6 months ( ± 7.04), the mean age of patients without OFC was 40 years and 5 months ( ± 13.1), and the mean age of patients without OFC was 32 years and 3 months (± 8.77).

**Table 1:** Demographic characteristics of all participants.

Variable	Control participants (N=69)	ABI patients without orbito frontal syndrome (OFS) (N=34)	ABI patients with orbito frontal syndrome (OFS) (N=10)	Significance
Age (M ± SD)	27.53 ± 7.04	40.41 ± 13.01	32.23 ± 8.78	p< 0.001
Country of birth (%)				p<0.01
-Israel	87.1	61.8	100	
-other	11.0	32.4	0	
Education Level (%)				p<0.001
-No high school diploma	0	4.0	30.0	
- Partial high school diploma	1.4	11.8	20.0	
- Full high school diploma	59.4	26.5	30.0	
- Undergraduate degree	21.7	23.5	20.0	
- Master's Degree or Higher	15.9	8.8	0	
Employment Status (%)				p<0.01
- Employed	52.2	8.8	10.0	
- Rehabilitative Employment	1.0	100	100	
In education	68.6	2.9	0	
ADHD Diagnosis (%)	21.7	35.3	40.0	p=.19
Marital Status (%)				p<0.01
- Single	71.0	38.2	50.0	
- Married	25.2	47.1	20.0	
- Other	1.9	8.8	30.0	
Smoking Status (Yes/No) (%)				p=.21
Smokers	15.9	20.6	40.0	
Non-Smokers	82.6	79.4	60.0	
Alcohol use (%)				p=.67
-Never	30.4	40.6	10.0	
-A few times per year	31.9	37.5	40.0	
-Once a month	23.2	18.8	30.0	
- Two to four times per week	8.7		10.0	
-Four to seven times per week	2.9	3.1	10.0	
Coffee Consumption (%)				p=.99
- 0 Cups	23.2	25.0	10.0	
- 0-1 Cups	13.0	25.0	30.0	
- 1-2 Cups	39.1	21.9	50.0	
- 3-4 Cups	20.3	21.9	10.0	
- 5-6 Cups	1.4	3.1	0	
- 7-9 Cups	2.9	3.1	0	
Psychiatric Medications Use (%)	4.3	23.5	30.0	p<0.01
Past Drug Use (%)	21.7	23.5	50.0	p=.18
Drug Use In the last week (%)	7.1	8.8	10.1	p=.58

### 2.2 Questionnaires

#### 2.2.1 Demographic questionnaire

The demographic questionnaire included the following items: age, country of birth, years of education, current occupation, marital status, and a report on substance use (such as cannabis, alcohol, and nicotine).

### 2.3 Excessive smartphone use

Excessive smartphone use was assessed using the Smartphone Addiction Scale - Short Version (SAS-SV; 49). This questionnaire, which is presented in Appendix No. 1, was developed to measure excessive smartphone use and is a shortened version of the original Smartphone Addiction Scale (SAS; 49). The SAS-SV consists of 10 items, and respondents rate their level of agreement with statements such as “I use my smartphone more than I intended” on a Likert scale ranging from 1 (strongly disagree) to 6 (strongly agree). The total score is the sum of all items and ranges from 10 to 60, with higher scores indicating more problematic and excessive smartphone use. Kwon et al. (49) reported high internal reliability for the SAS-SV (α=0.91), and the abbreviated questionnaire has shown a high correlation (over 0.7) with the original SAS and demonstrated high internal reliability in other studies (50). The questionnaire has been validated across different cultures and countries, including Israel (51), Morocco (52), Brazil (53), Spain, and Bulgaria (50). Furthermore, the questionnaire has been used across various age groups, including young and adult populations (53).

### 2.4 Obsessive–compulsive symptoms

The Yale–Brown Obsessive–Compulsive Scale (YBOCS; 54) was used in this study to assess obsessive–compulsive symptoms. The YBOCS is a 10-item self-report questionnaire that measures the severity of obsessive–compulsive symptoms. Participants rate their symptoms on a Likert scale ranging from 0 (not at all) to 4 (extremely). The total score is the sum of all items, and higher scores indicate more severe obsessive–compulsive symptoms. The YBOCS has good psychometric properties, including high internal reliability (α=0.89; 54).

### 2.5 Depression, anxiety, and stress

The Depression, Anxiety, and Stress Scale (DASS-21; 55) was used to assess symptoms of depression, anxiety, and stress. This 21-item self-report questionnaire is divided into three scales, with seven items per scale. Participants rated the extent to which each statement described their experiences in the past week on a four-point Likert scale (0=does not describe my situation at all, 3=describes my situation to a great extent).

The DASS-21 has strong psychometric properties, with high internal consistency across clinical and general populations (55); α=0.88 for anxiety, α=0.82 for depression, α=0.90 for stress, α=0.93 overall. Studies on excessive smartphone use also reported high internal reliability (Cronbach’s α above.90), as noted by Squires et al. (56) and Ali et al. (57). Additionally, the DASS-21 has demonstrated reliability and validity for assessing symptoms in individuals following ABI (58) and has shown strong correlations with other measures of depression and anxiety (59).

### 2.6 Trait impulsivity

The 15-item Barratt Impulsiveness Scale-short form (BIS-15), developed by Spinella (60) from the original BIS-11 (61), was used to assess trait impulsivity. The BIS-15 includes three subscales that assess different aspects of impulsive behavior. Lack of planning measures absence of future orientation (e.g., “I say things without thinking”). Motor impulsivity evaluates impulsive actions (e.g., “I do things without thinking”). Attentional impulsivity focuses on difficulties in sustaining attention (e.g., “I am restless in lectures or conversations”). Participants rate behaviors on a Likert scale from 1 (“never”) to 4 (“almost always”). Higher scores indicate greater impulsivity for each subscale. Previous studies support the scale’s high validity and reliability (62). For instance, it showed good internal reliability in assessing impulsivity among smartphone users, with motor impulsivity (α=0.82), attentional impulsivity (α=0.72), and lack of planning (α=0.80) (63). Additionally, individuals with ABI showed higher impulsivity levels compared to healthy participants (31).

### 2.7 Computerized tasks

#### 2.7.1 Impulsive choice—delayed discounting task

The Experiential Delay Discounting Task (EDT; 64) assesses impulsive behavior related to difficulties in delaying gratification. In the EDT, participants choose between a larger, delayed, uncertain monetary reward ($1.20) and a smaller, immediate, certain reward. The task comprises four blocks of 15 trials each, with different delay times (1, 5, 10, and 20 s) presented randomly across participants. For example, an EDT item can be $1.2.

The subjective value of delayed rewards influences an individual’s willingness to delay gratification. Therefore, the reduction in the subjective value of a future reward due to delay reflects delayed value. The EDT measures delay discounting, which represents the decrease in reward value when delayed compared to immediate availability. Choices and associated delay times are recorded as dependent variables to calculate this value (65). The area under the discounting curve (AUC) is used as the dependent variable, where lower AUC values indicate greater impulsiveness and lower self-control. Additionally, indifference points—which represent equal preference between two reward options—are used to assess the discount-delay gradient and isolate the impact of delay on value (66). See Weinstein et al. (32) for a detailed description of the EDT and its analysis. Individuals who play computer games excessively showed difficulties in delaying gratification compared to a control group (32). Similarly, individuals with ABI exhibited comparable difficulties relative to healthy participants (31).

#### 2.7.2 Impulsive action—inhibitory control—the Go/No-Go task

The Go/No-Go task is a computerized task that assesses inhibitory control (67). In the task, participants are prompted to respond to blue squares (go events) by pressing a button as quickly as possible. However, they must intentionally delay their response to orange squares (No-Go events). Stimuli were presented randomly for 100 ms, with an inter-stimulus interval of 2,000 ms. Commissions, which occur when participants respond to No-Go events, are considered an indicator of impulsivity. Omissions, which occur when participants fail to respond to Go events, are considered a measure of inattention. The task duration is 10 min and includes a training phase consisting of 10 steps. The number of commissions and omissions are dependent variables for this experiment.

### 2.8 Smartphone application

A smartphone application was used in this study to assess participants’ device usage patterns. Participants were requested to install the application on their smartphones. The application tracked the frequency of device unlocks and the duration of device usage, which were used as dependent variables. To ensure uniformity in the usage indicators among the subjects, all users of Android devices downloaded and activated the same application—Stay Free (https://stayfreeapps.com). Usage indicators were collected over a full week at two time points: at the first measurement and at the second measurement, which was carried out after 5 months. Since no suitable application was found for use on both Android and iPhone, iPhone users submitted the built-in usage reports available on the device. A preliminary test was performed to ensure that the measurement was consistent in nature with the measurement performed on Android. These objective data were particularly significant in the current study because individuals with brain injuries often experience deficits in self-awareness (68). Therefore, relying solely on self-report questionnaires may present methodological challenges.

### 2.9 Procedure

#### 2.9.1 Assessment and treatment

Individuals with brain injury and OFS were assigned to the group based on diagnostic assessments at the rehabilitation institute at baseline T1. These assessments included injury mechanism evaluation, post-injury brain imaging (CT or MRI), family member interviews, clinical interviews, neuropsychological test results, and completion of the Behavior Rating Inventory of Executive Function-Adult (BRIEF-A) by both the diagnosed individuals and a family member. The BRIEF-A, a standardized self and family member’s report assessment tool, evaluates executive functions and self-regulation in adults aged 18 to 90, screening for developmental, systemic, neurological, and psychiatric conditions, such as attention deficit disorders, traumatic head injuries, mild cognitive impairments, and dementia (69). These diagnostic findings enabled the assessment of whether the individuals exhibited symptoms meeting the criteria for OFS. Isolated frontal syndrome is uncommon, with most cases presenting mixed symptoms from multiple frontal regions (70). Additionally, brain injuries often involve multiple regions beyond the frontal lobe (71).

The rehabilitation program was based on a holistic approach specifically designed to facilitate optimal adjustment to the patients’ new post-injury reality, including within occupational contexts. The program is administered by a multidisciplinary team, predominantly composed of rehabilitation psychologists and neuropsychologists. The program addresses several key dimensions (46): (a) functional rehabilitation, which involves structured group attendance at the institute 4–5 days per week at regular, predefined times; (b) individual and group psychotherapy, focusing on issues such as changes in self-identity, depression, and anxiety; (c) cognitive interventions conducted both individually and in group settings, with an emphasis on increasing awareness of cognitive difficulties, psychoeducation regarding the implications of cognitive decline, acquisition of compensatory strategies and coping mechanisms, and cognitive training utilizing specialized software; (d) vocational preparation designed to facilitate reintegration into employment, including self-exploration, understanding occupational implications related to the injury, and assistance in identifying suitable vocational directions; and (e) guidance provided to family members who choose to participate. The program underscores the significance of occupational reintegration as a source of psychological wellbeing, meaning, and enhanced self-worth.

After receiving approval from the ethics committees of the university and the National Institute for Neuropsychological Rehabilitation, participants were recruited for this longitudinal study, which included two phases conducted at different times. Informed consent was obtained at the start at baseline T1, detailing the study’s purpose, voluntary participation, the right to withdraw at any time, and the measures taken to ensure data anonymity and confidentiality. Participants then completed demographic and mental wellbeing questionnaires, administered electronically via the Qualtrix platform to ensure anonymous data collection. Participants then completed computerized cognitive tasks. Subsequently, they installed a smartphone application to monitor device usage over 7 days, after which the data were submitted to the researcher. The second phase, conducted 5 months later at T2, replicated the same procedures.

### 2.10 Statistical and data analysis

The analysis of the results was performed on Statistical Package for Social Science (SPSS) for Windows v.21 (IBM Corp., Armonk, NY, USA).

### 2.11 Sample characteristics

Variables such as marital status, country of birth, employment, psychiatric medication use, and attention-deficit/hyperactivity disorder (ADHD) diagnosis were analyzed using chi-square tests. Continuous variables, including age, history of substance use, alcohol consumption, smoking, and coffee intake, were analyzed using one-way analysis of variance (ANOVA), with group serving as the independent variable. When significant group differences were identified, *post-hoc t*-tests with Bonferroni corrections were performed. A significance threshold of α=0.05 was defined for all results.

### 2.12 Behavioral and self-report measures

To examine group effects at baseline, analysis of covariance (ANCOVA) was conducted with all demographic and health measures used as covariates to assess delay discounting (DDT), inhibitory control (Go/No-Go task), self-report impulsivity, smartphone use (average daily hours of device use and the average daily number of unlocks), and measures of mental health questionnaires. *Post-hoc t*-tests with Bonferroni correction were applied in cases of significant group differences. To assess longitudinal changes, a repeated-measures ANOVA was used to evaluate group differences at baseline (T1) and 5 months later (T2), using the measures as in the baseline analysis. *Post-hoc t*-tests with Bonferroni correction for dependent samples were performed when a significant interaction between time and group type was observed. A significance threshold of α=0.05 was defined for all results.

## 3 Results

### 3.1 Between-group differences in demographic and health variables

Participants’ demographic data and drug use history are described in **Table 1**. The groups did not differ by past drug use, [*F*(2, 108) =1.75, *p*=1.78], drug use in the last week [*F*(2, 108) =0.54, *p*=0.58], alcohol use [*F*(2, 108) =1.75, *p*=1.78], smoking [*F*(2, 108) =1.60, *p*=2.01], coffee consumption [*F*(2, 108) =0.01, *p*=0.99], or past ADHD diagnosis (χ^2^ = 3.32, *p*=0.19). However, there were between-group differences in age [*F*(2, 108) =15.77, *p*<0.001], education level [*F*(2, 108) =8.61, *p*<0.001], country of birth (χ^2^ = 11.37, *p*<0.01), marital status (χ^2^ = 15.95, *p*<0.01), employment status (χ^2^ = 24.72, *p*<0.001), and psychiatric medication use (χ^2^=11.04, *p*<0.01). Given the significant group differences in certain demographic variables, these variables were examined as potential confounding factors, and ANCOVA analyses were conducted to control for these demographic variables.

### 3.2 Impulsivity, smartphone use, and mental health at baseline

#### 3.2.1 The Go/No-Go task

**Figure 1:**
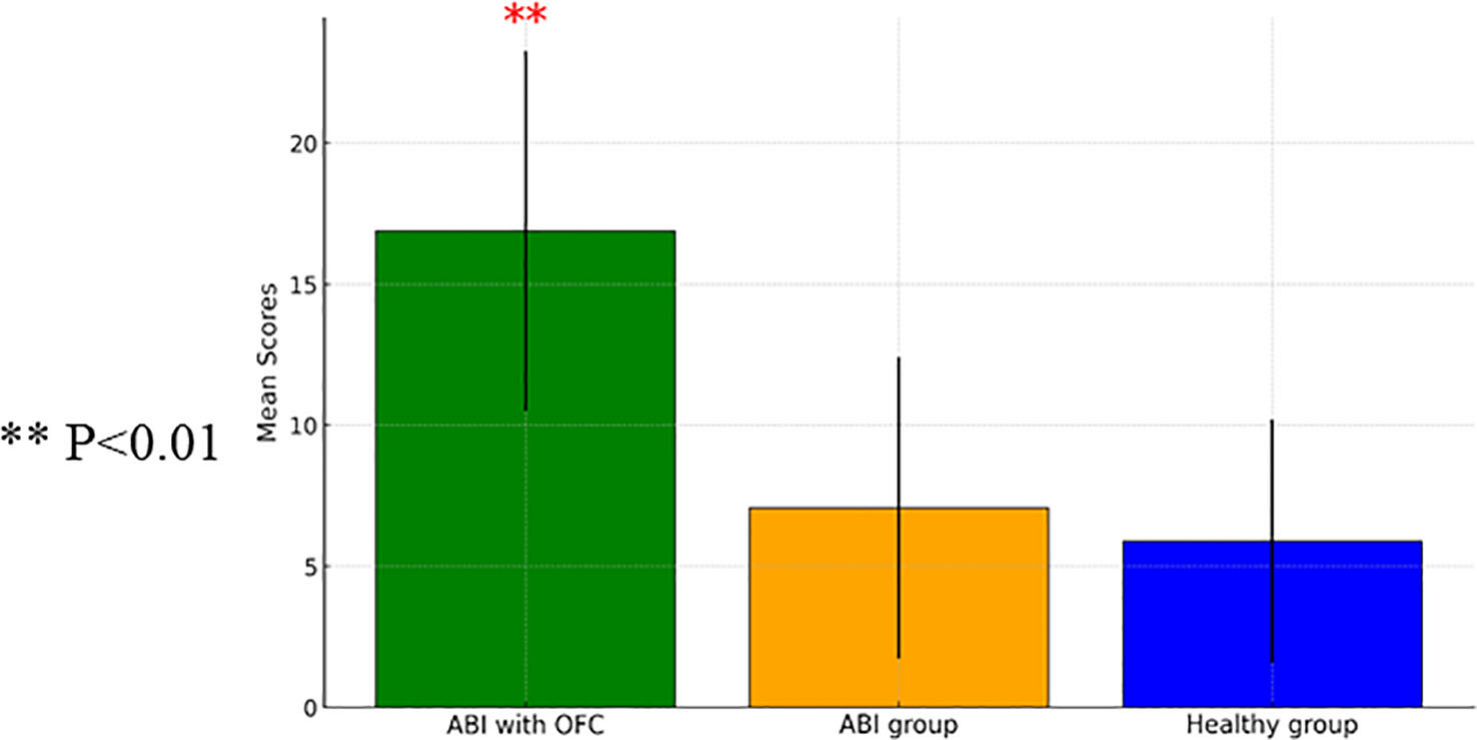
A one-way ANCOVA comparing commission mean scores measured on the Go/ no GO Task between Individuals with Acquired Brain Injury (ABI) (n=28), ABI with Orbito Frontal Syndrome (OFS) (n=10), and control participants (n=60) (with SD). ** p<0.01.

A one-way ANCOVA examining the average number of commission errors showed a significant group effect [*F*(2, 82)=9.84, *p*<0.001]. The ABI group with OFS exhibited greater impulsivity compared to both the healthy group and the ABI group without OFS, [*t*(60)=3.07, *p*<0.001; *t*(38)=8.73, *p*<0.001]. **Figure 1** shows a comparison between the ABI with OFS and the control group in commission errors on the Go/No Go task.

#### 3.2.2 Delay discounting task

An ANCOVA revealed a non-significant group effect [*F*(2, 30)=1.10, *p*=0.35], after controlling for all demographic variables.

#### 3.2.3 Self-reported trait impulsivity (BIS)

**Figure 2:**
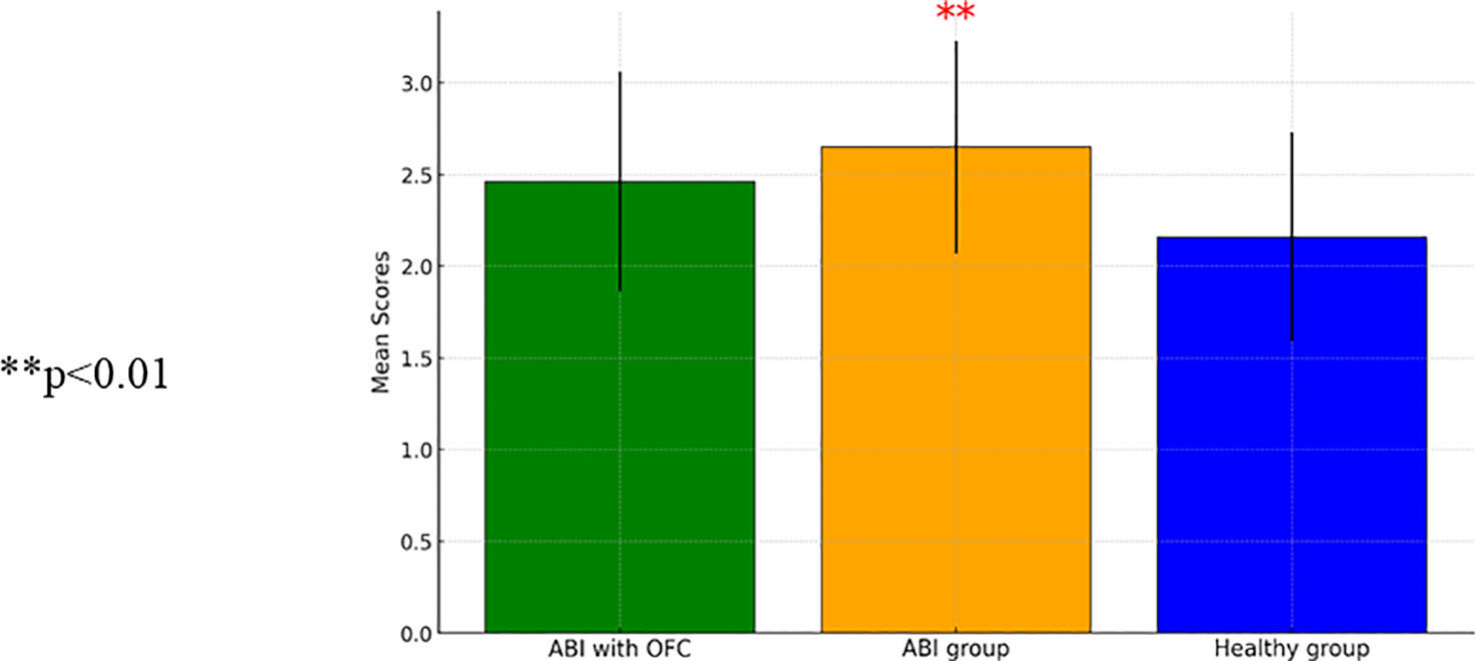
A repeated measure ANCOVA comparing attention impulsivity mean scores between patients with Acquired BARIN Injury (ABI) (N=32), patients with ABI and Orbito Frontal Syndrome (OFS) (n=10), and control participants (n=67) (SD). ** p<0.01.

The analyses of group differences in trait impulsivity using self-report questionnaires (BIS-11) showed mixed results. ANCOVA revealed non-significant group effects for motor impulsivity [*F*(2, 95)=1.20, *p*=0.31] and non-planning impulsivity [*F*(2, 95)=0.86, *p*=0.43]. However, ANCOVA for attention impulsivity showed a significant group effect [*F*(2, 95)=4.74, *p*=0.01], indicating higher attention impulsivity in individuals with ABI without OFS compared to healthy control participants [*t*(91)=−3.56, *p*<0.001]. **Figure 2** shows a comparison of attention impulsivity scores between patients with ABI, patients with ABI and OFS, and control participants.

#### 3.2.4 Excessive smartphone use

**Table 2** shows mean daily smartphone use in hours and mean daily number of unlocks in control participants, patients with ABI, and patients with ABI and OFS.

**Table 2:** A comparison of mean daily screen time and mean daily device unlocks in all participants- Mean (SD).

Variable	Patients with ABI (n=23)	Patients with ABI & OFS (n=9)	Control participants (n=64)
Mean Daily Screen Time	4.08 (1.92)	6.83 (2.12)	5.25 (2.18)
Mean Daily DeviceUnlocks	167.54 (109)	209.57 (126)	284.84 (182)

ABI, Acquired Brain Injury; OFS, Orbito-Frontal Syndrome.

**Figure 3:**
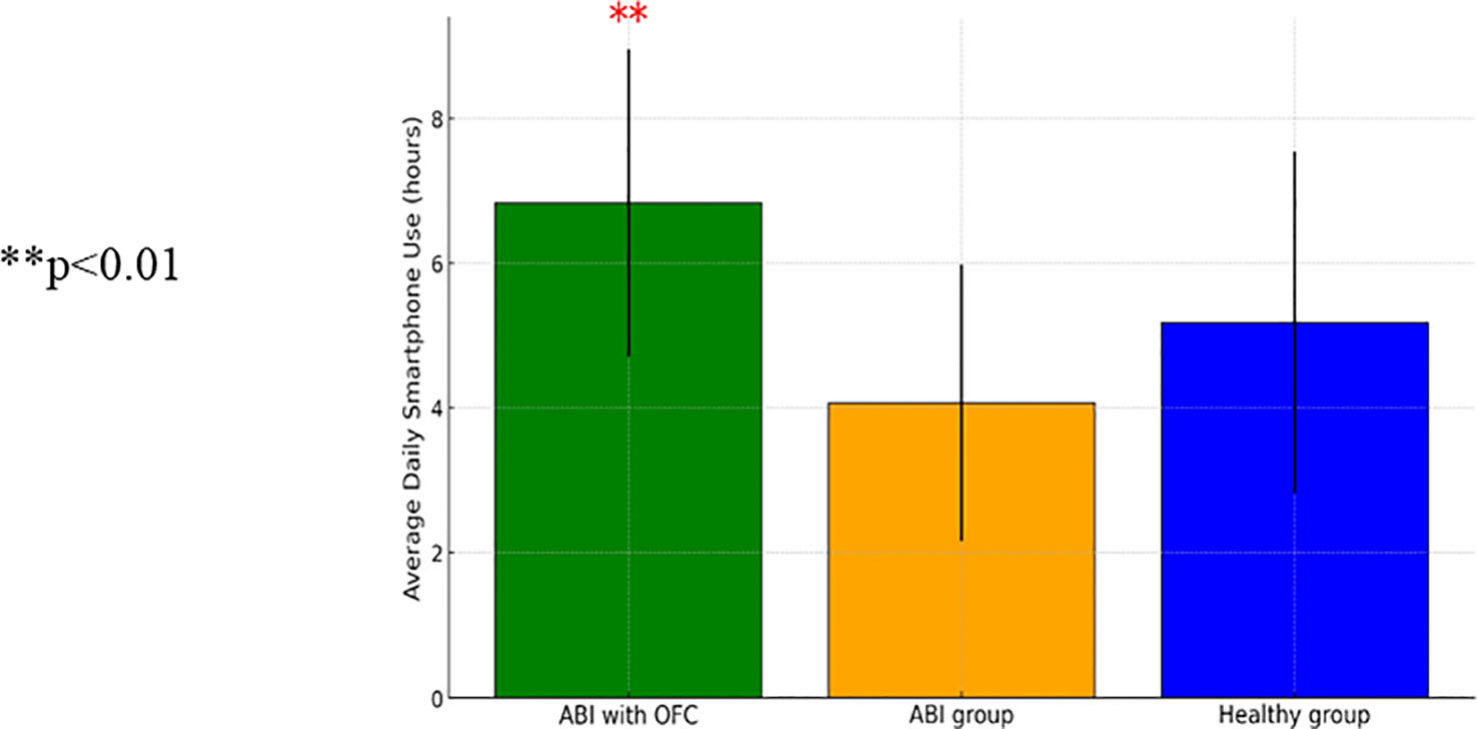
A one-way ANCOVA comparing average daily hours of device use between patients with Acquired Brain Injury (ABI) (n-64), patients with ABI with Orbito Frontal Syndrome (OFS) (n=9), and control participants (n=23) (SD) ** p<0.01..

ANCOVA analyses were conducted to examine excessive smartphone use, utilizing a usage tracking app that monitored the average daily hours of device use and the average daily number of unlocks, as well as a self-report questionnaire. The analysis revealed a significant group effect on average daily hour usage [*F*(2, 82)=5.29, *p*<0.01], and a significant effect of education level [*F*(1, 82)=11.68, *p*<0.01]. Participants with higher education had longer daily usage time compared to those with lower education [*t*(95)=−2.09, *p*<0.05]. Individuals with ABI and OFC showed significantly higher daily smartphone use in hours compared to both the control group [*t*(95)=−2.09, *p*<0.05] and the ABI group without OFS [*t*(86)=2.175, *p*<0.05]. **Figure 3** presents a comparison between individuals with ABI and OFS, the ABI group without OFS, and the control group in daily smartphone use.

An ANCOVA conducted on the average daily number of device unlocks did not reveal a significant group effect [*F*(2, 74)=1.76, *p*=0.18]. Similarly, the results of the self-report questionnaire (SAS) indicated no significant group differences [*F*(2, 95)=0.22, *p*=0.80].

#### 3.2.5 Mental health

To analyze differences in mental health variables between participants with ABI and control participants, a series of ANCOVAs was conducted. The analysis of depressive symptoms revealed a main effect of group, indicating that participants with ABI reported higher levels of depressive symptoms compared to control participants [*t*(109)=−3.17, *p*<0.01]. Age showed an effect on depression scores [*F*(1, 106)=6.00, *p*=0.02], indicating that older participants tended to report lower depression scores. Education, however, did not show an effect on depression scores [*F*(1, 106)=0.14, *p*=0.71]. No significant group differences were found for anxiety, stress, compulsive symptoms, or obsessive scores on the YBOCS. However, there was a trend *p*=0.07 for a group difference in compulsive symptoms. See **Table 3** for ANCOVA analyses comparing group differences on computerized tasks and mental health measures at baseline.

**Table 3:** Summary of ANCOVA analyses comparing group differences at baseline.

Measure	Group effect	Significance
Commission (Go/No-Go)	F(2, 82) = 9.84	**p < .001**
Delay of Gratification (K)	F(2, 30) = 1.10	p = .35
Motor Impulsivity (BIS-11)	F(2, 95) = 1.20	p = .31
Non-planning Impulsivity (BIS-11)	F(2, 95) = 0.86	p = .43
Attention Impulsivity (BIS-11)	F(2, 95) = 4.74	**p < .05**
Daily Device Use	F(2, 82) = 5.29	**p < .01**
Daily Number of Unlocks	F(2, 74) = 1.76	p = .18
Smartphone Addiction Scale (SAS)	F(2, 95) = 0.22	p = .80
Depression	F(1, 96) = 7.38	**p < .01**
Anxiety	F(1, 96) = 1.23	p = .27
Stress	F(1, 96) = 2.23	p = .14
YBOCS-compulsive	F(1, 96) =3.31	p = .0714
YBOCS Obsessive	F(1, 96) = 0.27	p = .61

ANCOVA, Analysis of Covariance; BIS-11, Barratt Impulsiveness Scale; YBOCS, The Yale-Brown Obsessive Compulsive scale.

Significant results in bold.

#### 3.2.6 Longitudinal changes in impulsivity, smartphone use, and mental health

A repeated-measures ANCOVA was conducted to assess changes in delay discounting employing the *K* measure over time. The results revealed no significant group effect [*F*(2, 18)=0.63, *p*=0.55], non-significant time effect [*F*(1, 18)=1.42, *p*=0.25], and no interaction effect between group and time [*F*(2, 78)=2.51, *p*=0.11]. A repeated-measures ANCOVA included all demographic and health factors. There was an interaction effect between time and group [*F*(2, 18)=3.85, *p*<0.05] but no significant group effect [*F*(2, 18)=0.43, *p*=0.66] or significant time effect [*F*(1, 18)=2.19, *p*=0.16].

**Figure 4:**
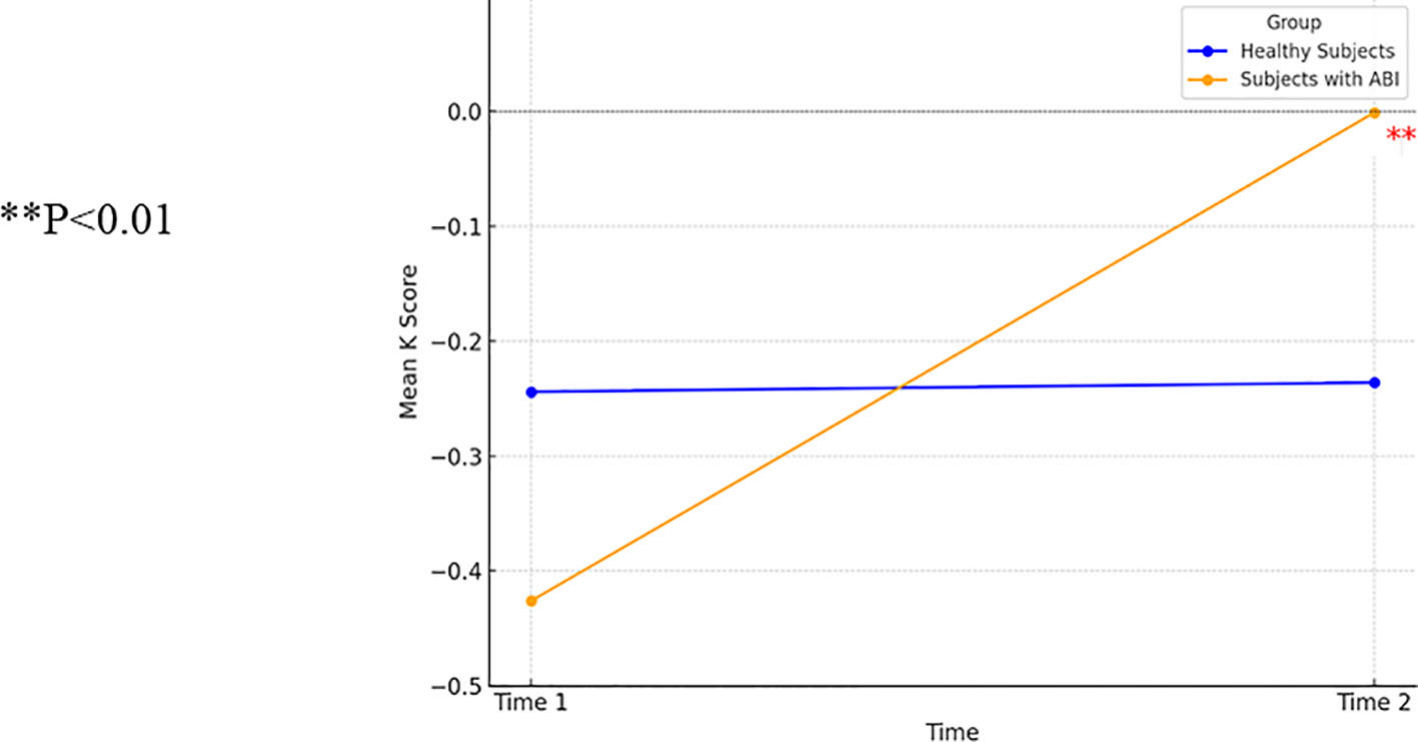
A one-way ANCOVA comparing delay discounting (K) in patients with ABI (n=9) and healthy control participants (n=23). ** p<0.01.

Because of an insufficient number of participants with OFS at the second measurement, the two ABI groups were combined, and all demographic and health factors were used as factors for co-variance. The repeated-measures ANCOVA demonstrated a group by *K* interaction [*F*(1, 19)=7.38, *p*<0.05], a non-significant effect of *K* [*F*(1, 19)=1.93, *p*=0.18], and a non-significant group effect [*F*(1, 19)=0.31, *p*=0.59]. Participants with ABI demonstrated improved delay discounting between the first and second measurements [*t*(8)=5.06, *p*<0.01]. Control participants showed no change between the first and second measurements [*t*(22)=−1.8, *p*=0.86]. **Figure 4** presents the interaction between time and group on delay discounting (*K*) from the repeated-measures ANOVA (two groups).

In the analysis of motor impulsivity, a repeated-measures ANCOVA revealed no group effect [*F*(2, 83)=0.26, *p*=0.77] or time effect [*F*(1, 83)=0.92, *p*=0.34]. A significant interaction between time and group was found [*F*(2, 83)=4.57, *p*<0.05]. *Post-hoc* analyses did not reveal significant changes over time within the groups: control participants [*t*(67)=0.51, *p*=0.61], patients with ABI [*t*(18)=−1.51, *p*=0.15], and patients with ABI with OFS [*t*(6)=1.98, *p*=0.09].

In commission errors, an ANCOVA showed a group effect [*F*(2, 70)=17.78, *p*<0.001], no effect of time [*F*(1, 70)=0, *p*=0.99], and no interaction between time and group [*F*(2, 70)=0.04, *p*=0.96]. *Post-hoc* comparisons indicated that participants with ABI-FS made more errors than control participants [*t*(60)=3.07, *p*<0.001] and those with ABI without frontal syndrome [*t*(38)=8.73, *p*<0.001]. *Post-hoc* comparisons indicated that participants with ABI-FS made more errors (M=16.88, SD=6.40) than both control participants (M=5.89, SD=4.31) [*t*(67)=6.39, *p*<0.001] and those with ABI without frontal syndrome (M=7.07, SD=5.35) [*t*(36)=4.43, *p*<0.001].

##### 3.2.6.1 Trait attention impulsivity

The ANCOVA showed no group effect [*F*(2, 78)=1.92, *p*=0.15], no effect of trait attention impulsivity [*F*(1, 78)=1.10, *p*=0.30], and no interaction between attention impulsivity and group [*F*(2, 78)=1.70, *p*=0.19]. No main effects of time, group, or interactions were found for the following variables: non-planning impulsivity, stress, mean daily device unlocks, excessive smartphone use (SAS scores), mean daily screen time, depression scores, anxiety scores, stress score, compulsive behavior scores, and obsessive behavior scores. **Table 4** shows a comparison of v*ariables assessing impulsivity, smartphone use, and mental health at baseline and follow-up.*

**Table 4A:** A repeated measures ANOVA for trait impulsivity, response inhibition, delay discounting, smartphone use, mental well-being, and obsessive-compulsive symptoms at baseline and follow-up.

Variable	Time effect	Group effect	Time x group interaction
Attention Impulsivity	F(1, 78) = 1.10, p = .30	F(2, 78) = 1.92, p = .15	F(2, 78) = 1.70, p = .19
Motor Impulsivity^1^	F(1, 83) = 0.92, p = .34	F(2, 83) = 0.26, p = .77	F(2, 83) = 4.57, **p < .05**
Non-planning Impulsivity	F(1, 91) = 13.11, p= .54	F(2, 91) = 1.47, p = .24	F(2, 91) = 1.35, p = .27
Commission Errors Go/No-Go task	F(1, 70) = 0.00, p = .99	F(2, 70) = 17.78, p < .001*	F(2, 70) = 0.04, p = .96
Delay Discounting (K)^2^	F(1, 19) = 1.93, p = .18	F(1, 19) = 0.31, p = .59	F(1, 19) = 7.38, **p < .05**
Mean Daily Screen Time	F(1, 70) = 0.04, p = .83	F(2, 70) = 1.52, p = .23	F(2, 70) = 0.18, p = .83
Mean Daily Device Unlocks	F(1, 62) = 2.09, p = .15	F(2, 62) = 2.35, p = .10	F(2, 62) = 1.18, p = .31
Smartphone Addiction Scale	F(1, 91) = 3.23, p = .08	F(2, 91) = 1.58, p = .21	F(2, 91) = 0.64, p = .53
Stress	F(1, 91) = 0.35, p = .55	F(2, 91) = 0.35, p = .71	F(2, 91) = 1.25, p = .29
Depression	F(1, 91) = 0.62, p = .43	F(2, 91) = 2.65, p = .08	F(2, 91) = 1.62, p = .20
Anxiety	F(1, 91) = 0.28, p = .60	F(2, 91) = 0.78, p = .46	F(2, 91) = 0.02, p = .98
YBOCS-Compulsive	F(1, 90) = 0.44, p = .51	F(2, 90) = 0.24, p = .80	F(2, 90) = 1.12, p = .33
YBOCS-Obsessive	F(1, 90) = 0.05, p = .82	F(2, 90) = 0.13, p = .88	F(2, 90) = 0.26, p = .77

^1^Post-hoc analyses did not reveal significant within-group differences over time.

^2^Participants with ABI demonstrated improved delay discounting between the first and second measurements [t (8) = 5.06, p < .01].

YBOCS- The Yale-Brown Obsessive Compulsive scale.

* p<0.05.

Significant results in bold.

**Table 4B:** A t-test comparison of trait impulsivity, response inhibition, delay discounting, smartphone use, mental well-being, and obsessive-compulsive symptoms before and after treatment in comparison groups.

ABI Group without Orbito Frontal Syndrome (OFS)				
Variable	Before mean (SD)	After mean (SD)	t(df)	p
Motor Impulsivity	2.07 (0.28)	2.16 (0.34)	t(18) = -1.509	.149
Non-Planning Impulsivity	3.21 (0.67)	3.35 (0.57)	t(18) = -0.908	.376
Attention Impulsivity	2.63 (0.63)	2.36 (0.54)	t(18) = 3.153	<.01**
Delay Discounting (K)	-0.47 (0.26)	-0.01 (0.14)	t(6) = -4.48	<.01**
Commission Errors Go/No-Go task	6.11 (4.57)	5.84 (4.23)	t(18) = 0.29	.77
Smartphone Addiction Scale	2.83 (0.95)	2.55 (0.81)	t(18) = 1.41	.17
Mean Daily Screen Time	4.31 (1.75)	4.43 (2.22)	t(11) = -0.36	.72
Mean Daily Device Unlocks	191.64 (106.64)	157.26 (83.52)	t(10) = -5.94	<.01**
Depression	1.65 (0.56)	1.88 (0.77)	t(18) = 2.08	<.05*
Stress	1.83 (0.63)	1.78 (0.71)	t(18) = 0.69	.48
Anxiety	1.49 (0.54)	1.44 (0.45)	t(18) = 0.41	.68
YBOCS - Obsessive	1.92 (0.85)	1.93 (0.82)	t(17) = -0.09	.93
YBOCS - Compulsive	1.64 (0.79)	1.42 (0.61)	t(17) = 1.59	.13
ABI Group with Orbitofrontal Syndrome (OFS)				
**Variable**	**Before Mean (SD)**	**After Mean (SD)**	**t(df)**	**p**
Motor Impulsivity (BIS-11)	2.34 (0.41)	1.97 (0.21)	t(6) = 1.98	.09
Non-Planning Impulsivity (BIS-11)	2.77 (0.83)	3.31 (0.61)	t(6) = -1.76	.13
Attention Impulsivity (BIS-11)	1.94 (0.68)	1.83 (1.10)	t(6) = 0.94	.38
Delay Discounting (K)	-2.98 (2.40)	0.10 (0.70)	t(1) = -2.49	.24
Commission Errors Go/No-Go task	16.33 (6.25)	16.50 (7.77)	t(5) = -0.08	.94
Smartphone Addiction Scale (SAS)	2.90 (0.70)	2.74 (0.49)	t(6) = 0.65	.55
Mean Daily Screen Time	6.05 (1.86)	6.31 (2.96)	t(5) = -0.27	.80
Mean Daily Device Unlocks	168.76 (89.33)	173.40 (82.93)	t(5) = -0.12	.91
Depression	1.59 (0.54)	1.51 (0.61)	t(6) = 0.33	.75
Stress	1.82 (0.72)	1.51 (0.51)	t(6) = 1.24	.26
Anxiety	1.47 (0.40)	1.43 (0.47)	t(6) = 0.44	.67
YBOCS - Obsessive	1.74 (0.68)	1.83 (1.10)	t(6) = -0.40	.70
YBOCS - Compulsive	1.46 (0.61)	1.51 (0.65)	t(6) = 0.40	.70

YBOCS, The Yale-Brown Obsessive Compulsive scale.

BIS-11 = Barratt Impulsiveness Scale. * p<0.05, ** p<0.01.

Significant results in bold.

Finally, the cutoff point of the SAS is 31. At baseline, the percentage of the patient group that met criteria for smartphone addiction was 39%, and in the control group, it was 54%. After the rehabilitation program, the percentage of the patient group that met criteria for smartphone addiction was 16%, and in the control group, it remained the same (54%). It should be noted that 12 out of the 17 patients who met criteria for smartphone addiction at baseline did not report their SAS scores after treatment; hence, there is no reliable evidence that the drop in percentage of smartphone addiction in this group is due to an improvement, as the data are missing.

## 4 Discussion

### 4.1 Major findings of impulsive action and trait attention impulsivity

The present study showed that patients with ABI and OFS exhibited higher impulsive action indicated by more commission errors in the Go/No-Go task. This evidence aligns with findings from Dimoska-Di Marco et al. (45), who reported impaired inhibitory control in patients with OFS. Secondly, patients with ABI without OFS showed higher trait attention-impulsivity ratings measured by the BIS-11 compared with control participants. This finding is compatible with prior findings of attentional deficits in patients with ABI (72, 73), which is likely associated with disruptions in the distributed neural networks underlying attentional control, indicated by the hypo-activation of a frontal area of the cognitive control network (left pre‐supplementary motor area) (74). There were no differences in trait ratings of motor and planning impulsivity and self-reported impulsivity measures between patients with ABI with OFS and control participants. The lack of differences may stem from impaired self-awareness due to frontal lobe injuries, affecting the reliability of self-report measures (75, 76) or possibly due to a small number of participants. Additionally, this may be due to the varying salience of impulsivity type post-ABI. Attention deficits are more prevalent and have an impact on daily function in ABI, making them more readily reported, while motor and planning impulsivity may be under-reported (77, 78). Finally, there was a trend of patients with ABI scoring higher on measures of compulsive symptoms. It is well established that measures of impulsivity and compulsivity are often correlated in behavioral addictions in general (79) and in excessive smartphone use, indicating that excessive smartphone use lies in the impulsive–compulsive spectrum.

### 4.2 Smartphone use

This study’s digital metrics, collected via a smartphone application, revealed that patients with ABI and OFS had higher daily smartphone usage duration compared to both patients with ABI without OFS and control participants, but there were no group differences in device unlock frequency. These findings may be attributed to executive function deficits, such as impaired cognitive shifting and difficulties in transitioning or disengaging from ongoing activities (21, 34). Rather than frequently unlocking their devices, patients with ABI and OFS tend to demonstrate prolonged usage sessions, which is likely affected by challenges in task-switching, response inhibition, and delayed gratification (41, 43). These findings suggest that executive dysfunction in patients with OFS manifests primarily as extended rather than frequent smartphone use, highlighting specific dispositional usage or inflexibility. It is also plausible that patients with ABI use the smartphone for social purposes as a compensation for loneliness and boredom. Healthy individuals make a more efficient and purposeful use of smartphones, since they are busy with other activities. There is evidence that individuals with excessive smartphone use often do so due to boredom and a desire for entertainment (3, 80). The lack of group differences in self-reported excessive use was due to the limitation in the accuracy of subjective tools in assessing smartphone use (81). This happens especially in patients with ABI, who may have impairments of self-awareness, limiting their ability to evaluate their usage patterns (68). Furthermore, patients with ABI showed higher depression scores, in accordance with previous research showing elevated rates of depression in patients with ABI (82, 83). The differences may be attributed to psychosocial challenges, such as changes in social roles, loss of independence, and cognitive difficulties (83).

### 4.3 Improved impulsive choice—delay discounting

This study revealed improvements in impulsive choice as shown by delay discounting among patients with ABI. Improvements in delay discounting highlighted the program’s specific benefit in treating decision-making in patients with ABI. These findings align with prior research on rehabilitation outcomes in ABI. Zucchella et al. (84) reported improvements in attention, as measured by the Trail Making Test (TMT), Attentive Matrices, and the Rey Auditory Verbal Learning Test (RAVLT), and executive function, measured by the Frontal Assessment Battery (FAB) in patients with ABI. Similarly, Gehring et al. (85) found cognitive improvements in glioma patients’ attention, memory, and executive functions, using the Stroop Color-Word Test, Digit Span Forward and Backward, and Visual Verbal Learning Test (VVLT). These studies support the efficacy of neuropsychological interventions in enhancing cognitive and executive functions. The occupational therapy, which prepared the patients for future employment, may have also improved their impulsive choice ability, indicated by an improvement in delayed discounting.

### 4.4 Impulsive action and mental wellbeing

The lack of improvement over time in impulsive action indicated by commission errors and the non-planning impulsivity trait ratings among patients with ABI may indicate deficits in impulsive action or inhibitory control deficits, during treatment (86, 87). Although there is evidence for the effectiveness of attention training after traumatic brain injury, modest improvement was also observed for non-treatment control groups (48). The absence of improvement in mental wellbeing among patients with ABI in treatment may be due to a lack of awareness (38, 88).

### 4.5 Limitations

This study has a small sample size of patients with ABI and OFS, which may have limited the detection of differences between patients. Classifying participants with OFS was challenging due to overlapping and diffuse brain injury. There was a small number of patients with OFS, and that limited the conclusions that can be drawn from the results of this sample. This limitation exists in studies that examine patients after brain injury, and because most brain injuries are widespread, they involve many brain regions and networks, and it is difficult to isolate the injury by region. It is not always possible to identify the exact brain region and the networks that were damaged. All patients participated in a pre-employment occupational rehabilitation program. Only patients whose cognitive ability and functional level were sufficient to successfully integrate into employment after rehabilitation were accepted into the study. Second, the study duration of 5 months may have been too short to detect changes, particularly in smartphone use habits. Third, self-report measures may be biased due to impaired self-awareness, especially for participants with OFS. Fourth, there were significant differences in age and education between groups. Although the ANCOVA controlled for these variables, these differences may have affected the results. Fifth, there is concern that problematic smartphone usage may be more significantly correlated with the proportion of specific smartphone usage purposes than with overall smartphone usage time. Sixth, we used the *K* measure to differentiate delay discounting between groups. However, it is highly recommended to compute the AUC for the Delay Discounting Task, rather than relying solely on the discounting parameter *K*. AUC provides a model-free, standardized measure of impulsive choice and has been consistently linked to orbitofrontal cortex functioning, which is highly relevant for the present ABI population. Finally, no external psychiatric evaluation was conducted for the control participants, and it was essentially done by using self-reported questionnaires. The criterion for attention deficit disorder was also established by self-report, and not by clinical diagnosis. No intervention was performed for the control group.

### 4.6 Conclusions

Patients with ABI and OFS showed higher measures of impulsive action and longer smartphone use compared with patients with ABI without OFS and control participants, and they were more depressed than control participants. Occupational rehabilitation treatment improved impulsive choice, which was indicated by improved delay discounting. This improvement is probably due to training patients in control of their impulsivity, as part of occupational treatment before returning to work. This study highlighted the heightened risk for excessive smartphone use in patients with ABI, particularly in those with OFS, indicating the importance of early identification and tailored prevention enhancing behavioral change. Future research should include larger samples, longer follow-up, and objective measures to support self-reports. Advanced brain imaging could improve diagnostic accuracy and insights into neurological changes as a result of treatment.

## Data Availability

The raw data supporting the conclusions of this article will be made available by the authors, without undue reservation.
